# Effects of Mogrosides on High-Fat-Diet-Induced Obesity and Nonalcoholic Fatty Liver Disease in Mice

**DOI:** 10.3390/molecules23081894

**Published:** 2018-07-29

**Authors:** Xiaobing Zhang, Yunfei Song, Yipei Ding, Wei Wang, Ling Liao, Jin Zhong, Pengbo Sun, Fan Lei, Yaou Zhang, Weidong Xie

**Affiliations:** 1 Department of Chemistry, Tsinghua University, Beijing 100084, China; zxb17@mails.tsinghua.edu.cn (X.Z.); 15139915181@163.com (Y.D.); 2 State Key Laboratory of Chemical Oncogenomics, Graduate School at Shenzhen, Tsinghua University, Shenzhen, China; zhangyo@sz.tsinghua.edu.cn; 3 Guilin Layn Natural Ingredients Corp., Guilin 541100, China; song.yunfei@layn.com.cn; 4 Open FIESTA Center, Tsinghua University, Shenzhen 518055, China; kappa33wei@live.com (W.W.) 15602927350@163.com (L.L.); zhongjin1996@163.com (J.Z.); pb_sun@foxmail.com (P.S.); 5 Key Lab in Healthy Science and Technology, Division of Life Science, Graduate School at Shenzhen; Tsinghua University, Shenzhen 518055, China; 6 Laboratory of Pharmaceutical Science, School of Life Science, School of Medicine, Tsinghua University, Beijing 100084, China; lifn@163.com

**Keywords:** Siraitia grosvenorii, mogrosides, obesity, nonalcoholic fatty liver disease, AMPK, p62

## Abstract

Obesity and nonalcoholic fatty liver disease (NAFLD) are highly prevalent and cause numerous metabolic diseases. However, drugs for the prevention and treatment of obesity and NAFLD remain unavailable. In this study, we investigated the effects of mogrosides (luo han guo, LH) in *Siraitia grosvenorii* saponins on high-fat-diet-induced obesity and NAFLD in mice. We found that compared with the negative control, LH reduced body and liver weight. LH also decreased fat accumulation and increased AMP-activated protein kinase (AMPK) phosphorylation (pAMPK) levels in mouse livers. We also found that high-purity mogroside V upregulated pAMPK expression in HepG2 cells. In addition, high-purity mogroside V inhibited reactive oxygen species production and upregulated sequestosome-1 (SQSTM1, p62) expression in THP-1 cells. These results suggest that LH may affect obesity and NAFLD by enhancing fat metabolism and antioxidative defenses. Mogroside V may be a main component of LH. However, the exact molecular mechanisms and active components responsible for the inhibitory effects of LH on obesity and NAFLD require further investigation.

## 1. Introduction

Obesity is defined as abnormal or excessive fat accumulation or a body mass index ≥30 kg/m [1]. Obesity rates in low- and middle-income countries are increasing. One-third of the approximately 2 billion individuals who are overweight are considered obese [2]. Obesity exerts severe health effects, degrades life quality, and imposes a massive social burden. However, ideal drugs for the prevention and treatment of obesity are currently unavailable.

Nonalcoholic fatty liver disease (NAFLD) is the most common chronic liver disease. It may progress through the stages of simple bland steatosis, nonalcoholic steatohepatitis, hepatic fibrosis, and cirrhosis to hepatocellular carcinoma [3]. NAFLD is highly prevalent among overweight and obese individuals [4], and a definitive pharmacotherapy for NAFLD remains nonexistent. 

Individuals with obesity and NAFLD often consume a high proportion of fat [5,6]. The consumption of carbohydrates and sugar-sweetened beverages has been linked with adult and childhood obesity and NAFLD [7,8]. Sufficient scientific evidence suggests that decreasing the consumption of sugar-sweetened beverages will reduce the prevalence of obesity and obesity-related diseases [9]. Therefore, there is a large demand for sugar substitutes with high sweetness and low caloric content, especially among obese individuals.

Mogrosides (luo han guo, LH) are the main active components of *Siraitia grosvenorii* saponins, which are 200–350 times sweeter than sucrose [10]. LH has been extensively used in beverages owing to its high sweetness and nontoxicity. However, whether LH affects obesity and NAFLD remains to be determined. 

In this study, we investigated the effects of LH on high-fat-diet-induced obesity and NAFLD in mice. Moreover, we preliminarily analyzed the active mechanisms underlying the effects of mogrosides.

## 2. Results

### 2.1. Effects of LH on Biochemical Parameters 

**Figure 1:**
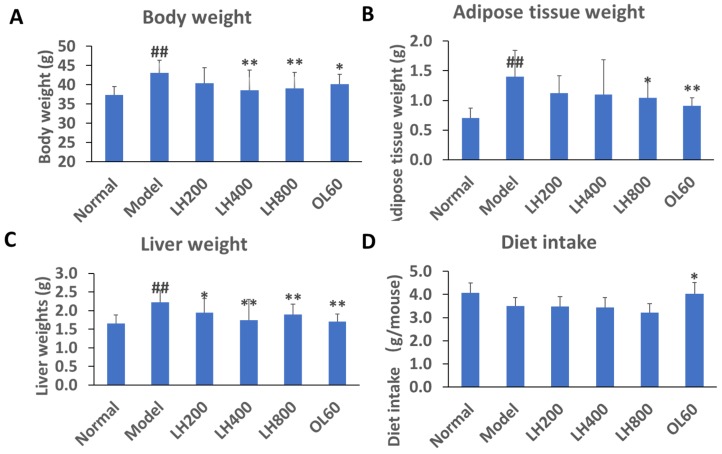
Effects of mogrosides (luo han guo, LH) on (**A**) body, (**B**) abdominal adipose, and (**C**) liver tissue weight and (**D**) diet intake of mice. Data are presented mean±SD, ^##^ *P* < 0.01 vs normal group; * *P* < 0.05 and ** *P* < 0.01 vs. high-fat control group, n = 10. Normal, normal group; model, high-fat control group. LH200, LH400, and LH800: groups orally treated with LH at dosages of 200, 400, and 800 mg/kg/day, respectively; OL60: group orally treated with Orlistat (OL) at 60 mg/kg/day.

After 4 weeks on a high-fat diet, mice in the high-fat control group had higher body weight than mice in the normal group (Figure 1A). However, the body weight of mice in the treatment groups treated with LH at dosages of 400 and 800 mg/kg/day was lower than that of mice in the high-fat control group. The body weight of mice in the 60 mg/kg/day Orlistat (OL) group was lower than that of mice in the high-fat control group. 

The weight of abdominal adipose tissues of mice in the high-fat control group was higher than that of mice in the normal group (Figure 1B). The weight of abdominal adipose tissues of mice in the 800 mg/kg/day LH group and 60 mg/kg/day OL group was lower than that of mice in the high-fat control group.

The liver weight of mice in the high-fat control group was significantly higher than that of mice in the normal group (Figure 1C); mice in the 200, 400, and 800 mg/kg/day LH groups; and mice in the 60 mg/kg/day OL group.

The food intake of the LH groups and the high-fat control group at 24 h after LH treatment did not significantly differ (Figure 1D). However, the food intake of the OL group at 24 h after OL treatment was significantly higher than that of the high-fat control group.

### 2.2. Effects of LH on Blood Glucose, Lipid, and Fecal TG Levels 

**Figure 2:**
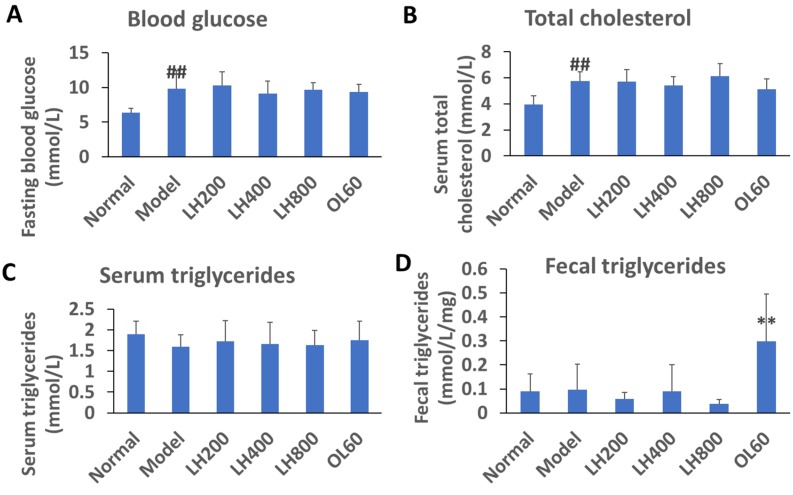
Effects of LH on (**A**) blood glucose, (**B, C**) lipid, and (**D**) fecal triglyceride (TG) levels of mice. Data are presented as mean ±SD, ^##^ *P* < 0.01 vs normal group; ** *P* < 0.01 vs high-fat control group, n = 10. Normal, normal group; model, high-fat control group. LH200, LH400, and LH800: groups orally treated with LH at dosages of 200, 400, and 800 mg/kg/day, respectively; OL60: group orally treated with OL at 60 mg/kg/day.

After 4 weeks on a high-fat diet, the fasting blood glucose and cholesterol levels of the high-fat control group were higher than those of the normal group; however, blood triglyceride (TG) levels of the high-fat control group did not change (Figure 2A–C). Moreover, the blood glucose and lipid levels of mice did not change after 4 weeks of treatment with orally administered OL. Compared with the normal group, the fecal TG content of the high-fat control did not increase. Fecal TG content was unaffected by LH treatment but increased under OL treatment.

### 2.3. Effects of LH on Abdominal Adipose Tissue Size, Liver Morphology, and Pathology 

**Figure 3:**
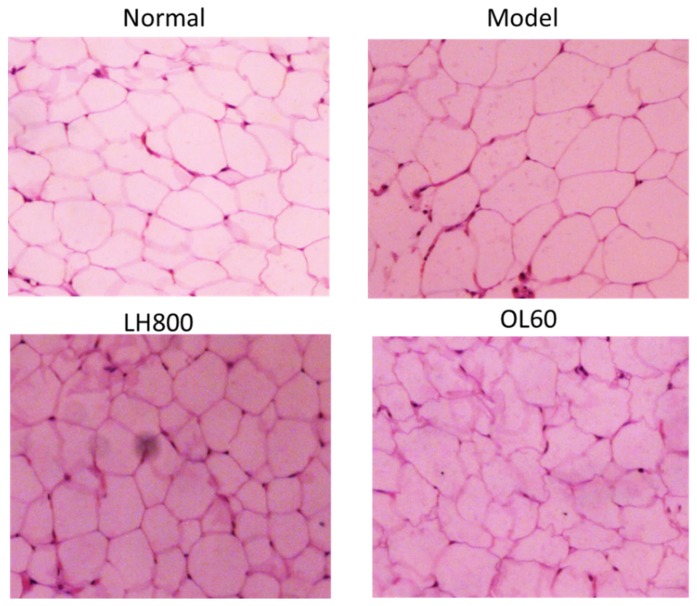
Effects of LH on the size of abdominal adipocytes of mice. Hematoxylin and eosin (H&E) staining (100× magnification). Normal, normal group; model, high-fat control group. LH800: group orally treated with LH at dosage of 800 mg/kg/day; OL60: group orally treated with OL at 60 mg/kg/day (n = 10).

After 4 weeks on a high-fat diet, the size of abdominal adipocytes of the high-fat control group increased compared with that of the normal group, as inferred from pathological slices. Given that treatment with 800 mg/kg/day of LH decreased abdominal adipose tissue, this dosage group was selected for further investigation. Compared with the size of abdominal adipocytes from the high-fat control group, the size from the 800 mg/kg/day LH group and 60 mg/kg OL group significantly decreased (Figure 3).

**Figure 4:**
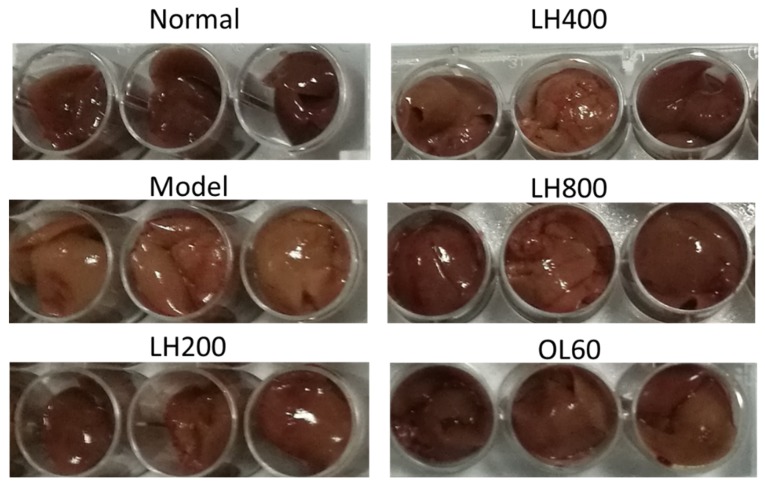
Effects of LH on the color of mouse livers. Normal, normal group; model, high-fat control group. LH200, LH400, and LH800: groups orally treated with LH at dosages of 200, 400, and 800 mg/kg/day, respectively; OL60: group orally treated with OL at 60 mg/kg/day (n = 10).

Meanwhile, the liver color of the high-fat control group appeared light yellow and that of the normal group was dark red. The liver color of groups treated with different dosages of LH and OL changed from light yellow to dark red (Figure 4).

**Figure 5:**
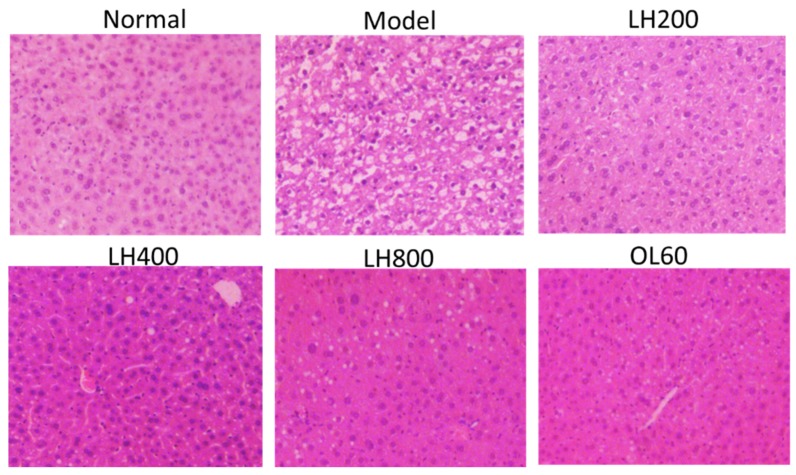
Effects of LH on the liver pathology of mice. H&E staining (100× magnification). Normal, normal group; model, high-fat control group. LH200, LH400, and LH800: groups orally treated with LH at dosages of 200, 400, and 800 mg/kg/day, respectively; OL60: group orally treated with OL at 60 mg/kg/day (n = 10).

Furthermore, histopathology analysis revealed that mice in the high-fat control group showed remarkable liver steatosis (Figure 5). However, fat accumulation decreased in mice in groups that were treated with different dosages of LH and OL.

### 2.4. Effects of LH on Serum ALT and AST Activity

**Figure 6:**
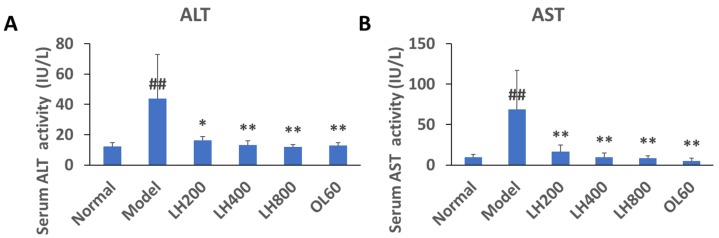
Effects of LH on (**A**) serum alanine aminotransferase (ALT) and (**B**) aspartate aminotransferase (AST) activity in mice. Data are presented as mean±SD, ^##^*P* < 0.01 vs normal group; * *P* < 0.05 and ** *P* < 0.01 vs. high-fat control group, n = 10. Normal, normal group; model, high-fat control group. LH200, LH400, and LH800: groups orally treated with LH at dosages of 200, 400, and 800 mg/kg/day, respectively; OL60: group orally treated with OL at 60 mg/kg/day.

Serum alanine aminotransferase (ALT) and aspartate aminotransferase (AST) activity increased in mice fed high-glucose and high-fat diets (Figure 6A,B). However, this increase was significantly attenuated by treatment with different dosages of LH and OL.

### 2.5. Effects of LH on Liver AMPK Phosphorylation 

**Figure 7:**
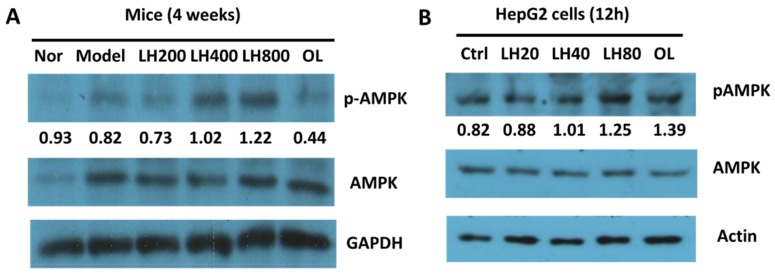
Effects of LH on pAMPK expression in **(A)** mouse livers and **(B)** HepG2 cells. Nor, normal group; model, high-fat control group; Ctrl, control. LH200, LH400, and LH800: groups orally treated with LH at dosages of 200, 400, and 800 mg/kg/day, respectively; OL60: group orally treated with OL at 60 mg/kg/day (n = 10). LH20, LH40, and LH80: LH (high pure mogroside V) at final concentrations of 20, 40, and 80 µg/mL, respectively (n = 3).

pAMPK expression was upregulated in the livers of mice that received 200–800 mg/kg LH orally (Figure 7A). Meanwhile, pAMPK expression levels increased in HepG2 cells incubated with 20–80 µg/mL high pure mogroside V, one of the main components of LH (Figure 7B). 

### 2.6. Effects of LH on Antioxidative Defenses in THP-1 Cells

**Figure 8:**
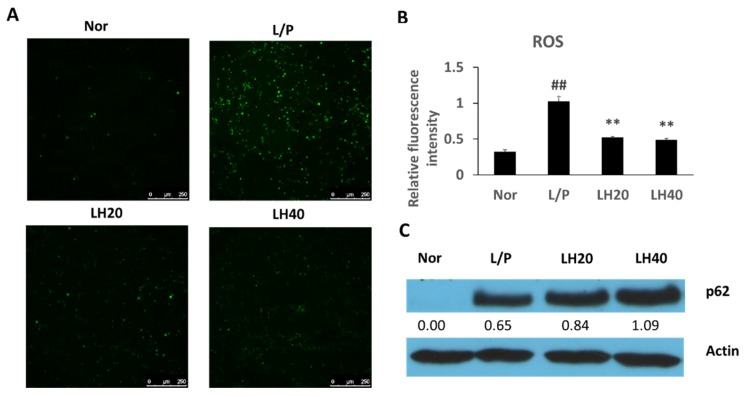
(**A,B**) Effects of LH on reactive oxygen species (ROS) and (**C**) p62 levels in THP-1 cells. Nor, normal control; L/P, LPS- and PMA-induced control group. LH20 and LH40: LPS- and PMA-induced groups simultaneously treated with LH (high pure mogroside V) at final concentrations of 20 and 40 µg/mL, respectively. Data are presented as mean ±SD, ^##^ *P* < 0.01 vs. normal control group; ** *P* <0.01 vs LPS- and PMA-induced control group (n = 3).

Reactive oxygen species (ROS) production increased in lipopolysaccharide (LPS)- and phorbol-12-myristate-13-acetate (PMA)-induced THP-1 cells and was inhibited by mogroside V treatment (Figure 8A,B). Hence, p62 expression in LPS- and PMA-induced THP-1 cells was assayed (Figure 8C). Compared with the control treatment, mogroside V treatment increased p62 expression. The above results prove that mogroside V can promote the activation of antioxidative defenses.

## 3. Discussion

An important tactic in the prevention or treatment of weight gain and obesity is usually inhibiting dietary intake and consumption. However, many drugs for curing obesity have been withdrawn because of serious side effects [11]. OL as a positive drug reduced gut fat absorption by inhibiting the activity of pancreatic lipase, but was still little used [12]. The results of this study provide preliminary evidence that LH inhibits obesity and fatty liver induced by a high-fat diet. The antiobesity effect of LH does not appear to be associated with decreased dietary intake or decreased fat absorption by the gut. Considering that we tested the antiobesity effect of LH in mice only within 1 month, a longer term of effect or safety evaluation needs to be further investigated in the future. 

LH may protect against the development of NAFLD by inhibiting fat droplet formation in the liver. NAFLD is associated with increased liver size, and hepatomegaly may be associated with hepatocyte enlargement caused by lipid accumulation [13]. Elevated serum AST and ALT activity indicates that liver cells may have been subjected to inflammatory damage [14]. Increased fat accumulation, inflammation, and oxidative stress in the liver may contribute to the development of NAFLD [15]. Actually, NAFLD is a complicated disease. Hormone, growth factor, and adipokine imbalance also plays an important role in the development of NAFLD [16]. AMPK is an important factor in the development of NAFLD, because its phosphorylation is responsible for the activation of fatty acid oxidation and energy metabolism [17]. Promoting AMPK activity is a viable therapeutic strategy for NAFLD [18]. The present work shows that mogroside treatment upregulates pAMPK expression in mouse livers. This effect was further validated in HepG2 cells. These results suggest that LH promotes fat oxidation metabolism to inhibit the development of NAFLD. 

LH exhibited anti-inflammatory effects in LPS-induced mice [19] and inhibited ROS production in LPS- and PMA-induced THP-1 cells. LPS and PMA increased ROS production in monocytes and macrophage cells [20,21]. NAFLD is associated with macrophage recruitment and consequent inflammation after adipocyte enlargement [22]. Oxidative stress also plays an important role in the development of NAFLD [23]. Immune cells, instead of liver cells, may contribute to the development of inflammation or oxidative stress in liver tissues. p62 is an important antioxidative defense factor that is also involved in autophagy [24]. In THP-1 cells, LH considerably increased p62 expression, although LPS combined with PMA also compensatively increased p62 expression. These results indicate that the potential effect of LH on oxidative stress or inflammation in livers may be associated with the increased capacity for antioxidative defense through the upregulation of p62 expression. 

LH inhibits obesity and NAFLD. The action mechanisms underlying this inhibitory effect may be associated with the promotion of AMPK phosphorylation and enhancement of antioxidative defenses through the upregulation of p62 expression. Mogroside V may be an active component of LH. LH does not affect blood glucose and lipid levels despite its sweet taste. Hence, it might be used as a natural alternative sweetener in food products for patients with NAFLD, obesity, diabetes, or hyperlipidemia. Nevertheless, the exact molecular mechanisms underlying the antiobesity and anti-NAFLD action of LH require further investigation.

## 4. Materials and Methods

### 4.1. Chemicals and Reagents

**Figure 9:**
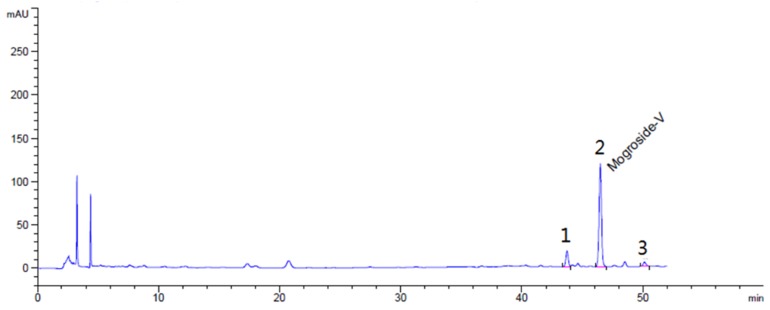
HPLC of Siraitia grosvenorii LH. Peak 1, 11-oxo-mogroside V, 6.8% (*g*/*g*); peak 2, mogroside V, 46% (*g*/*g*); peak 3, mogroside IV, 3.2% *g*/*g*.

LH (approximately 46% mogroside V, 6.8% 11-oxo-mogroside V, 3.2% mogroside IV, *g*/*g*) was purchased from Guilin Layn Natural Ingredients Corp. (Guilin, China). HPLC of LH is shown in Figure 9. High-purity mogroside V (98%) was obtained from Dasf Biotechnology Co., Ltd. (Nanjing, China). OL was purchased from Zhejiang Hisun Pharmaceutical Co., Ltd. (Taizhou, China). Glucose, total cholesterol (TC), and TG kits were purchased from BioSino Bio-Technology & Science Inc. (Beijing, China). ALT and AST kits were obtained from Nanjing Jiancheng Institute of Biotechnology (Nanjing, China). Bradford Protein Assay Kit was purchased from Beyotime Research Institute of Biotechnology (Shanghai, China).

### 4.2. High-Fat-Diet-Induced Mice

Sixty 4-week-old male NIH mice weighing 18–22 g were purchased from Guangdong Medical Laboratory Animal Center (Guangzhou, China). The animals were maintained in a room at a temperature of 20 ± 2 °C and humidity of 60% ± 5% under a 12 h dark/light cycle. All experiments were performed strictly in accordance with the recommendations of the Guide for the Care and Use of Laboratory Animals of the Institutional Animal Care and Use Committee of Tsinghua University (13_LQF3). The mice were randomly divided into 6 groups (n = 10 per group): normal, high-fat control, low-dose LH, medium-dose LH, high-dose LH, and positive. The normal group received a normal chow diet (weight ratio: 4% fat, 65% carbohydrates, 20% protein, *g*/*g* [25]; 10% of total calories from fat; 3.58 Kcal/g diet; Guangdong Medical Animal Center, Foshan, China), and the remaining groups received high-fat diets (weight ratio: 20% fat, 50% carbohydrates, 21% protein, *g*/*g*; 41% of total calories from fat; 4.7 Kcal/g diet; H10141; Beijing HFK Bioscience Co., Ltd., Beijing, China). LH was orally administered to the 3 LH groups at dosages of 200, 400, and 800 mg/kg/day. OL, the positive control, was orally administered to the positive control group at a dosage of 60 mg/kg/day. LH and OL were freshly prepared by dissolution in deionized water. Equal volumes of deionized water were fed to the normal and high-fat control groups. The food and water intake of the animals within 24 h of drug administration was observed twice a week. In the third week of treatment, fecal samples were collected and stored at −80 °C until assayed. After 4 weeks, the mice were fasted from 9:00 a.m. to 3:00 p.m. and anesthetized by intraperitoneal injection with 10% urethane solution (g/mL, dissolved in normal saline; Sangon Biotech, Shanghai, China) at a dosage of 10 mL/kg. Blood samples were collected from the orbital plexus. Serum samples were obtained from the collected blood samples through 5 min of centrifugation (5000 rpm, 4 °C) and stored at −20 °C until assayed. Liver and abdominal adipose tissues were rapidly removed and weighed. Portions of the liver and abdominal adipose tissue were stored in 4% paraformaldehyde solution for conventional hematoxylin and eosin (H&E) staining and slice analysis, and the remaining tissues were stored at −80°C for further biochemical analysis.

### 4.3. Biochemical Analysis

Blood glucose, TG, TC, AST, and ALT levels were assayed in accordance with the protocols provided with commercial kits. Feces (approximately 100 mg) was collected from each mouse, placed in 1.5 mL Eppendorf tubes, and dried in an oven at 60 °C for 30 min. Then, the dried feces was soaked for 30 min in 200 µL of PBS. The feces was homogenized in 800 µL of chloroform–methanol mixture (2:1, *v*/*v*). The homogenates were maintained for 5 min at room temperature and then centrifuged for 5 min at 12,000× *g* and 4 °C. Subsequently, 50 µL of the supernatants was transferred into Eppendorf tubes and dried in an oven for 1 h at 40 °C. The supernatants were incubated for 30–60 min in 250 µL of the working solution included with the TG kit. Finally, TG levels were quantified.

### 4.4. Cell Culture

HepG2 and THP-1 cells were provided by the Cell Resource Center of the Shanghai Institute for Biological Sciences, Chinese Academy of Sciences, Shanghai, China. Both cell lines were cultured in Dulbecco’s Modified Eagle’s Medium (high glucose, Gibco^®^, Thermo Fisher Scientific, Waltham, MA, USA) supplemented with 10% fetal bovine serum (FBS; Premium, Pan Biotech, Aidenbach, Germany) and 1% pen–strep antibiotics (Gibco™, Thermo Fisher Scientific, USA). Then, the cell lines were incubated in a humidified atmosphere at 37 °C with 5% CO_2_. The cells were seeded into 6-well plates at a density of 2.5 × 10^5^ cells per well. 

We assayed the cytotoxicity of high-purity mogroside V, one of the main components of LH, in HepG2 and THP-1 cells. Mogroside V at final concentrations of not more than 100 µg/mL within 48 h of incubation did not show any significant cytotoxic response on (3-(4,5-dimethyl-2-thiazolyl)-2,5-diphenyl-2-*H*-tetrazolium bromide (MTT) assay (MTT cell proliferation and cytotoxicity assay kit, Nanjing Jiancheng Bioengineering Institute, Nanjing, China). HepG2 cells were used to investigate the effects of high-purity mogroside V on pAMPK expression in mouse livers. After 12 h of attachment, HepG2 cells were incubated for 12 h with mogroside V at final concentrations of 20–80 µg/mL, and cell lysates were collected for Western blot analysis. 

The effects of high-purity mogroside V treatment on reactive oxygen species (ROS) production were investigated in THP-1 cells. Cells were incubated for 12 h with mogroside V at final concentrations of 20 and 40 µg/mL. Cell lysates were collected for further Western blot analysis.

### 4.5. ROS Assay 

The ROS assay was conducted in accordance with previous methods [20]. In this assay, 2,7-dichlorodi-hydrofluorescein diacetate (DCFH-DA) fluorescence probes were transported into cells and then hydrolyzed into DCFH. Intracellular active oxygen binds to DCFH and causes it to emit fluorescence that can be detected by fluorescence microscopy (excitation wavelength 485 nm; emission wavelength 525 nm). Here, THP-1 cells (5 × 10^4^ per well) were seeded into 24-well plates and incubated for 12 h. Subsequently, mogroside V was added at final concentrations of 20–40 µg/mL. After 30 min of incubation, the cells were incubated for 6–24 h with LPS (2 µg/mL final concentration) and PMA (200 ng/mL final concentration). Subsequently, the medium in each plate was replaced with fresh FBS-free medium containing DCFH-DA (10 mM final concentration), and the plates were incubated for 20 min. Then, the cells were washed thrice with fresh FBS-free medium and immediately observed under fluorescence microscopy at 200× magnification (Leica DMI6000B, Heidelberg, Germany). Six random areas with the same size were selected from the captured images of each sample. The gray density of fluorescence intensity in each area was calculated using ImageJ software (version 1.48, National Institutes of Health, Bethesda, MD, USA), and the average value of each sample was obtained from 3 biological replicates for further statistical analysis. The average gray density values of fluorescence intensity from the negative controls treated with LPS and PMA were defined as 1. All other values were normalized on the basis of this value.

### 4.6. Western Blot Analysis

Approximately 100 mg of liver tissue from each mouse was dissolved in 1 mL of cell lysate (50 mM Tris–HCl, 4 M urea, and 1% Triton X-100, pH 8.0). Western blot analysis was conducted with liver homogenates (approximately 50 µg of the protein samples). Protein levels were quantified by using the Bradford Protein Assay Kit. Target proteins were separated via SDS-PAGE electrophoresis and then transferred from gels to PVDF membranes under 220 mA for 1.5 h. After blocking with 5% bovine serum albumin for 1.5 h, the membranes were incubated overnight with primary antibodies at 4 °C. The primary antibodies included mouse polyclonal β-actin antibody (1:5000, A1978; Sigma-Aldrich Co., St. Louis, MO, USA) or rabbit polyclonal antibodies against GAPDH (1:2000, Cat. No. ABS118936A; Absin Bioscience Inc., Beijing, China), rabbit antibodies against AMPK and phospho-AMPKα (Thr172) (1:1000, Cat. No. 2532 and 2535, respectively; Cell Signaling Technology, Inc., Danvers, MA, USA), and p62 (1:2000, PM045; Medical & Biological Laboratories Co., Ltd., Nagoya, Japan). After incubation, the membranes were washed 4 times with Tris-buffered saline with Tween 20 (TBST) for 10 min per wash. Next, the membranes were incubated for 1.5 h with secondary antibodies, including horseradish–peroxidase-labeled goat anti-rabbit lgG H&L (1:5000, Cat. No. 074-1506; SeraCare/KPL Company, Milford, MA, USA), at room temperature. After incubation, the membranes were washed 5 times with TBST for 10 min per wash. The expression levels of target proteins were detected by using Pierce™ ECL Western Blotting Substrate (Cat. No. 32106, Thermo Fisher Scientific, USA). Protein levels were normalized to GAPDH or actin levels, and protein quantities were analyzed on the basis of relative gray density values by using ImageJ software (version 1.48, National Institutes of Health, Bethesda, MD, USA).

### 4.7. Statistical Analysis

Data are expressed as mean ±SD. Statistical significance was evaluated through one-way ANOVA and set as *P* < 0.05. When appropriate, Newman–Keuls comparison was used to determine the source of significant differences.
